# Supplementary material for: Faster Atlantic currents drive poleward expansion of temperate phytoplankton in the Arctic Ocean
Source: Nat Commun. 2020 Apr 6;11:1705. doi: 10.1038/s41467-020-15485-5 (PMC7136244; doi:10.1038/s41467-020-15485-5)
Supplement: Supplementary file 1 — Supplementary information [file 41467_2020_15485_MOESM1_ESM.pdf]

Supplementary Information for:

Faster Atlantic currents drive poleward expansion of temperate phytoplankton in the Arctic Ocean

Oziel L.[1,2*a], A. Baudena[3a], M. Ardyna[4,5], P. Massicotte[2], A. Randelhoff[2], J-B Sallée[3], R. B. Ingvaldsen[6], E. Devred[1], M. Babin[2]

Affiliations:
[1]Ocean and Ecosystem Sciences Division, Bedford Institute of Oceanography, Fisheries and Oceans Canada P.O. Box 1006, Dartmouth, Nova Scotia, Canada B2Y 4A2
[2]Takuvik Joint International Laboratory, Laval University (Canada) - CNRS (France), Département de biologie et Québec-Océan, Université Laval, Québec, Québec, G1V 0A6, Canada
[3]Sorbonne Université (UPMC Univ Paris 6)-CNRS-IRD-MNHN, LOCEAN-IPSL, 4 Place Jussieu, Paris F-75005, France
[4]Sorbonne Université & CNRS, Laboratoire d'Océanographie de Villefranche, 06230 Villefranche-sur-mer, F-06300, France
[5]Department of Earth System Science, Stanford University, Stanford, CA, 94305, USA
[6]Institute of Marine Research, N5817 Bergen, Norway.
[a]now at: Sorbonne Université & CNRS, Laboratoire d'Océanographie de Villefranche, 06230 Villefranche-sur-mer, F-06300, France

correspondence to: laurent.oziel@obs-vlfr.fr

This file includes:
Supplementary Methods (1)
Supplementary Notes (1-10)
Supplementary Figures 1 to 17
Supplementary Tables 1 to 4
References

1. Supplementary Methods

1.1. The 'leading-edge' detection method

The leading-edge is the main metric used in this study to illustrate the poleward (i.e. northward and eastward) expansion of the *Ehux* bloom. In a previous study[1], the leading-edge was defined as the maximum latitude reached by PIC pixels exceeding 0.006 mol L^{-1} in three distinct meridional areas (20-30°E, 30-40°E and 40-50°E). Note that for an optimal data coverage, summer PIC was considered as the maximum concentration of the July-August-September period[1]. Most of the spatial variability (i.e. expansion) of the *Ehux* bloom occurred in the eastern Barents Sea where the bathymetric control weakens and where the polar front variability is maximum[2,3]. Our study, by mixing Lagrangian experiments, Ocean color PIC data, considering both longitudinal and latitudinal shifts, required a more statistically consistent and reproducible method and an extended definition for the eastern Barents Sea.

First, the 'eastern Barents Sea' had to be defined in order to capture the southern Atlantic current branch. The purpose of focusing on the southern Atlantic inflow is to consider longitudinal shift as well as and to avoid re-circulations south of the central bank. A mask was made according to those constraints (the mask is available for download, see data availability section) and represents what we further consider the 'eastern Barents Sea'. The 'eastern Barents Sea' is the area east of the green line in **Supplementary Figure 1**. The leading-edge detection automatically excludes virtual particles outside this newly defined 'eastern the Barents Sea'.

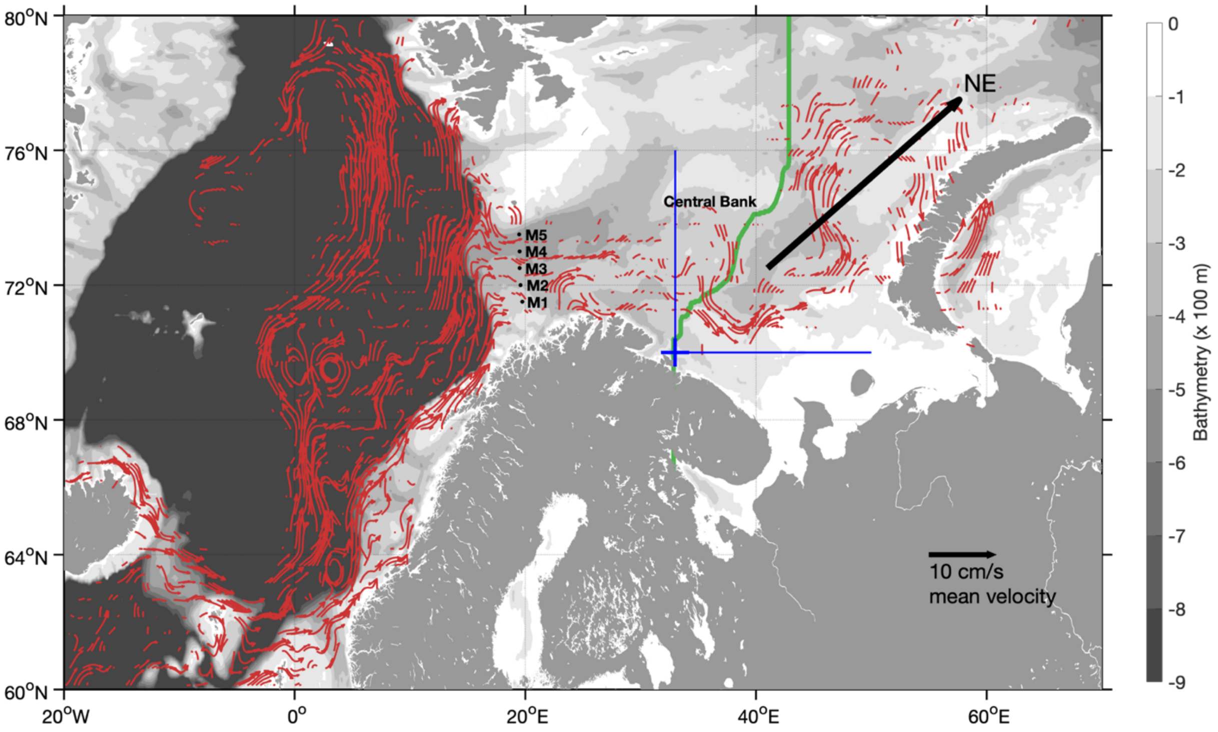

Supplementary Figure 1. Mean currents (from Mean Dynamic Topography, MDT-CLS13) associated with Atlantic Waters superimposed on bathymetry in gray palette. The eastern Barents Sea area used for the leading-edge detection is delimited by the green line. The reference fixed point (33°E-70°N) from which the leading-edge distance is derived is illustrated by the blue cross. This reference corresponds to the southwestern most position of the northeastward inflow of Atlantic Waters in the eastern Barents Sea area. The black arrow shows the North-East direction. The 5 mooring positions at the Barents Sea Opening (BSO) are indicated and labeled from M1 to M5.

Subsequently, we detected the leading-edge position as the crossing of the 95th percentile in latitude and in longitude of the virtual particles entering the eastern Barents Sea (**Supplementary Figure 2**). Therefore, the leading-edge does not necessarily correspond to a virtual particle position. For ocean color PIC data, particles were considered as pixels associated with PIC concentrations exceeding 0.002 mol L^{-1}. Finally, the distance corresponding to the expansion is derived as the distance between the leading-edge and a fixed position located at 33°E-70°N (see red cross in **Supplementary Figure 2**). This reference has been chosen given that it is the southwestern most position of the Atlantic current in the previously defined eastern Barents Sea. Few particles, however, ended up south of 70°N but were still considered (they generally represented less than 1% of the total amount).

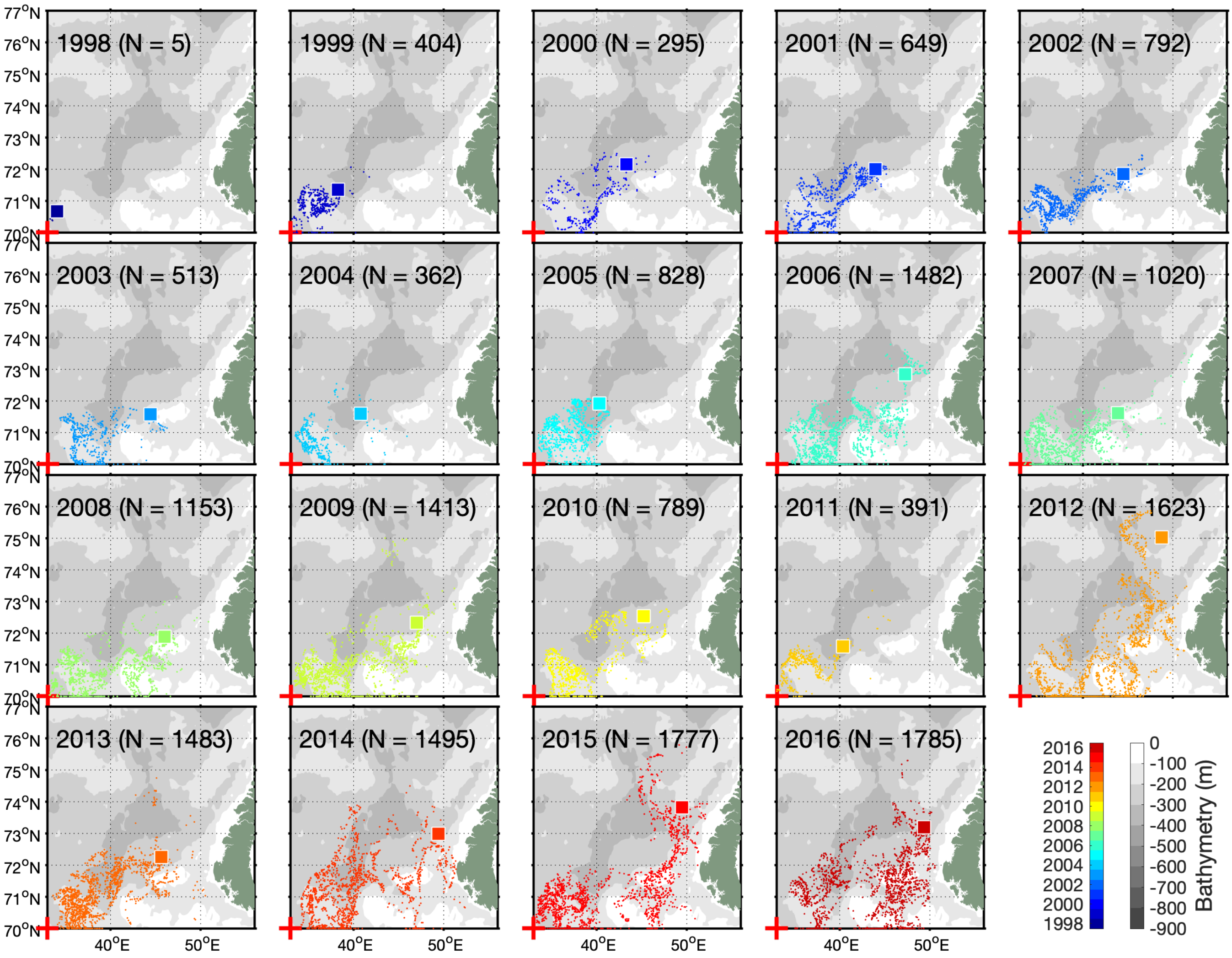

Supplementary Figure 2. Leading-edge (square) detection for the 1st Lagrangian experiment. The expansion of the leading-edge is expressed as its distance from the fixed point illustrated by a red cross (33°E-70°N).

2. Supplementary Notes

2.1. Validation of satellite-derived geostrophic velocities toward *in situ* instruments at BSO

To investigate the reliability of the satellite altimetry-derived velocity field, the satellite-derived geostrophic velocities were compared with *in situ* current meter data at 50 m depth from moorings between 71°30 and 73°30'N at the Barents Sea Opening (BSO, see in **Supplementary Figure 1**, time-series updated from[4]). Note that data from moorings are daily averaged to remove diurnal and semidiurnal tidal cycle, then weekly (7-days) averaged. Following the same method, 7-days composites maps from satellite altimetry data were produced, and time series were extracted averaging pixels within a 0.1° radius around the mooring location. In addition, a 4-weeks running mean were applied to both time series before deriving the statistics. The time series showed relatively similar varying current speed (Pearson's correlation coefficient R=0.43-0.71) and direction (**Supplementary Figure 3**). Overall, the geostrophic velocities derived from altimetry were generally underestimated in agreement with previous estimations[5].

No trend is evident in the BSO time-series from *in situ* current meter measurements or satellite-derived velocities extracted at the mooring's positions (**Supplementary Figure 3**). This is consistent with the maps of geostrophic derived fields showing no, or only weak trends in the southern and central BSO (**Figure 3** in the main text). The fact that the trend in the central BSO evidenced from the altimeter data is not captured by the presented time series (both the satellite-derived velocities at mooring's locations and the *in situ* current measurements) might partly be due to the length of the *in situ* time-series (these time series start in 1997). More importantly, according to the satellite-derived velocity fields, the strongest trend in the BSO is in the far northern part, which is not covered by the *in situ* instruments (**Supplementary Figure 4**). Episodic inflow events of Atlantic Water in this northern region have already been documented[6] and such events seem to have increased in frequency[7], adding support to the geostrophic derived trends in this region.

Note also that although geostrophic velocities caused by sea level changes associated with Ekman transports are important drivers for the Atlantic Water velocity field[8], there is also a baroclinic component of the flow associated with the internal density gradients[9]. Reliable estimation of the Atlantic Water flow in the full water column can be obtained from altimeter data, but then in combination with hydrography[10]. Such an approach is beyond the scope of this work focusing on transport of *Emiliania huxleyi* occurring in the upper water layers. Thus, the presented altimeter derived geostrophic velocity fields are to be taken as representative of the surface/upper layer flow of Atlantic Water and not for the full water column.

We note that recently, CMEMS is distributing a dataset produced by the Centre National d'Études Spatiales (CNES) and the Collecte Localisation Satellites (CLS) in the framework of the Globcurrent ESA project (http://www.globcurrent.org/) that delivers satellite-derived geostrophic velocities together with modelled Ekman transports and Stokes drift. A future inclusion of the Ekman and Stokes components could further improve the estimation of ocean currents and reduce errors[11] and should be considered for further studies. Alternatively, the European Space Agency Climate Change Initiative (CCI) project on "Sea Level" has reprocessed altimeter data to provide satellite-based sea level products to study climate-scale variations of sea level globally[12] and in the coastal zone[13]. The global product (available via ESA) provides monthly means of Mean Sea Level Anomaly 1/4° during the 1993-2015 period using TOPEX/Poseidon, Jason-1, Jason-2, ERS-1, ERS-2, GeoSat Follow-On (GFO), Envisat, SARAL/AltiKa and CryoSat-2 with special care of editing, cross-calibration, homogeneous

corrections, removal of global and regional biases, homogenization of long-spatial-scale errors, monthly optimal interpolation gridding. The need for daily temporal resolution for the Lagrangian experiments hindered us to use this data set.

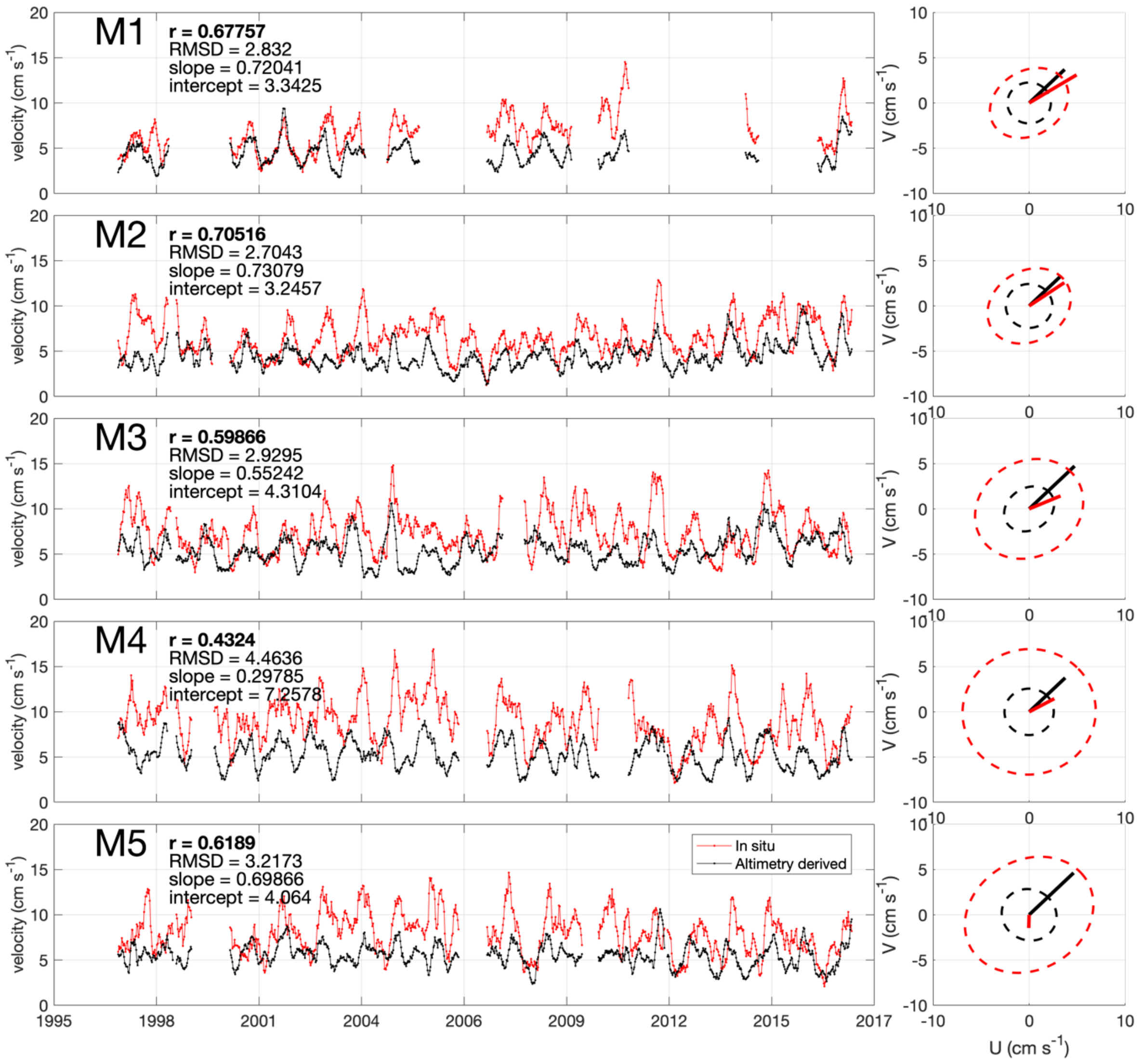

Supplementary Figure 3. Validation of satellite-derived geostrophic velocities towards in-situ velocities at 50 m deep from moored current meters at BSO (a hydrographic section located at the western entrance of the Barents Sea). Current meter data are daily averaged to remove tidal signal, then weekly averaged like satellite data. Moorings from M5 to M1 are plotted against derived geostrophic velocities (top to bottom) on the left panels, and associated variance ellipses and mean current velocity arrows are shown in the right panels. See map in **Supplementary Figure 1** for mooring positions. Statistics are the Pearson's correlation coefficient (r), Root mean squared difference (RMSD), and slope and intercept of the linear regression analysis.

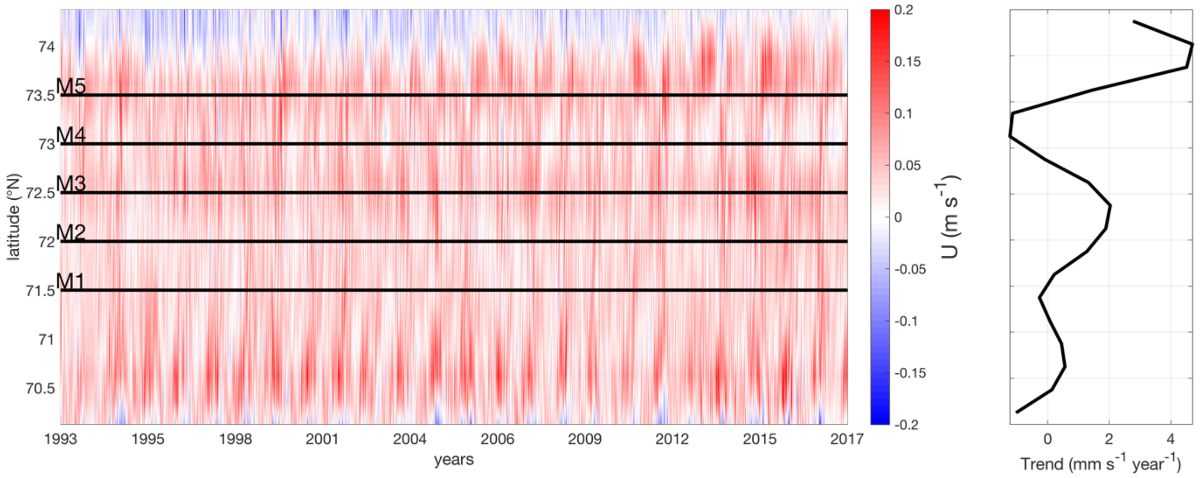

Supplementary Figure 4. HovMöller diagram of zonal geostrophic velocities (U, m s^{-1}) along latitudes (y-axis). Zonal geostrophic velocities trends (mm s^{-1} year^{-1}) against latitudes are illustrated in the right panel. The higher increase in the geostrophic velocity field occurs north of the northernmost current meter M5 and therefore explain why moorings are not capturing most of the trend observed with satellites. Mooring positions are illustrated by horizontal black lines. The reader can also refer to the map in **Supplementary Figure 1**.

2.2. Estimation of the steric effect versus mass related effect on geostrophic velocities

The regional variability of sea level is an integral indicator of changing oceanographic conditions. Sea level changes can be due to different processes of oceanic, atmospheric, or terrestrial origin[14]. However, the non-seasonal and interannual variability of sea level in the Barents Sea was found to be essentially driven by mass-related effects[15], i.e. sea water accumulation or spreading due to currents, and not by steric effects (contraction or expansion of seawater due to changes in salinity or temperature). The non-seasonal mass-related changes of sea level are responsible for a large part (up to 50%) of the non-seasonal sea level variability in the BS[15]. However, the SLA variability attributed to the steric and/or the advection is re-evaluated in this study to confirm previous estimates. This is achieved by using an in-situ hydrographical time series located at the western entrance of the Barents Sea Opening (BSO, meridional transect from 70°N to 74.5°N along the longitude 18°E). The sea level changes are due to (1) the divergence of water mass (mass effect/currents) and (2) the contraction or expansion of water column because of the changes in its density (steric effect). Thus, the total SLA measured by altimetry (SLA_T) can be decomposed in two components: the SLA due to thermo- or halo-steric effects (SLA_S) and SLA attributed to the mass-related effects (SLA_M). Satellite altimetry measurements, corrected for the inverted barometer effect, provide estimates of SLA_T, which are the sum of the mass-induced (SLA_M) and the steric (SLA_S) sea level anomalies (SLA_T=SLA_M+SLA_S). To investigate the role of steric effects in the variability of sea level in the EAC, we use the vertical profiles of temperature and salinity from hydrography data (World Ocean Database 13) to compute SLA_S following the equation:

$$\mathrm{SLA_S} = \mathrm{SLA_{TS}} + \mathrm{SLA_{HS}} = -\rho_0^{-1}\left(\int_{-H}^{0} \rho\,(T,\bar{S},z)dz + \int_{-H}^{0} \rho\,(\bar{T},S,z)dz\right)$$

where ρ_0=1027.5 kg m^{-3} is a reference density and $\bar{T}$ and $\bar{S}$ are the time-averaged values of T and S. The separation between thermosteric (TS) and halosteric (HS) heights is approximate due

to the nonlinear nature of the equation of state. The integration is performed over the entire depth range. The non-seasonal SLA in the satellite altimetry data is obtained by subtracting the 1993–2016 monthly mean climatology. Results from this study agree with previous estimations[15] (**Supplementary Figure 5**) with an overall yearly contribution of the steric effect of about 25-40%, the long-term increasing trend being primarily due to the mass effect (> 60 %, i.e. currents).

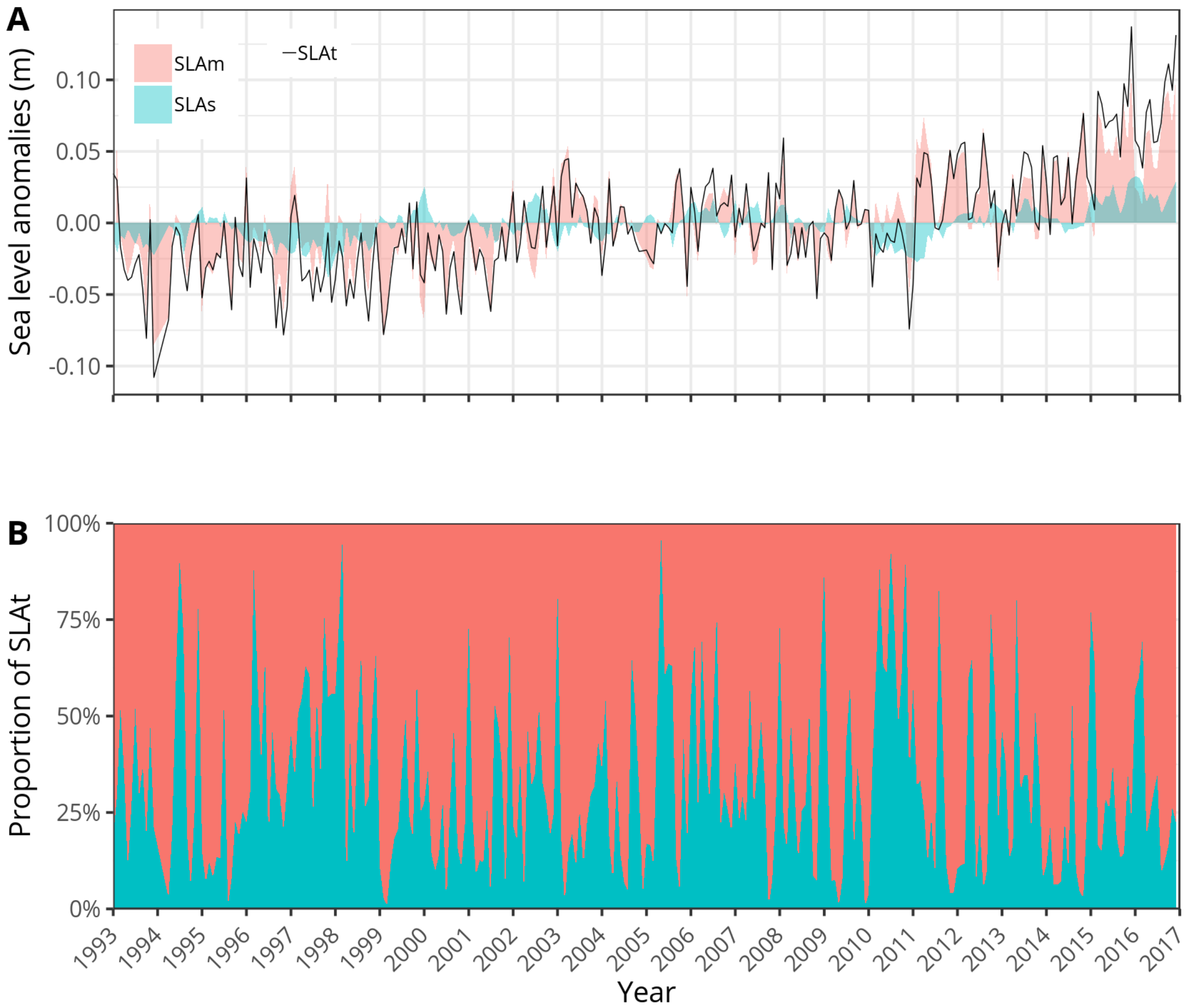

Supplementary Figure 5. (A) Sea Level Anomaly (SLA) derived from: satellite altimetry (SLAt or SLA total), hydrographic section time series at BSO (SLAs or SLA due to the steric effect), the difference of SLAt-SLAs=SLAm or SLA due to mass effect. (B) The relative contribution of SLAs and SLAm in SLAt variability.

2.3. The EOF and the relative trend in current velocities

Because Empirical Orthogonal Function (EOF) cannot deal with missing data and because high-frequency and seasonal variability is not the scope of this study, EOF was run on monthly non-seasonal Sea Level Anomaly (SLA) fields. However, some gaps corresponding to ice-covered areas (>15% in sea ice concentration) remained in the SLA fields and have been systematically filled with zeros. Thus, the EOF results of the study area do not consider the winter variability associated with high latitude ice-covered areas (i.e. the northern Barents Sea, Kara Sea and the Greenland Sea). This is why the quality of the EOF in the northern and eastern parts of the EAC is quite poor due to low data coverage and is manually masked. However, this

issue does not alter the Atlantic Water current monitoring in this study since the Atlantic domain is constantly ice-free.

Supplementary Table 1: Obtained explained variance for the first four EOF variability modes.

EOF	explained variance (%)
1	53.7
2	28.1
3	11.8
4	6.3

The EOF analysis on the non-seasonal SLA (1993-2016, **Supplementary Figure 6**) showed that the two first modes of variability accounted for more than 80% of the non-seasonal variability (**Supplementary Table 1**; respectively 53.7% and 28%). The sign of the spatial pattern of the first mode was consistent over the entire basin, with a peak in magnitude along the coasts and in the path of the AW. The time-series of the first mode revealed high energy at the decadal time scale, as indicated by its power spectrum (>10 years, **Supplementary Figure 7**). In contrast, the second mode exhibited a dipole structure of opposite signs between the Barents and Norwegian Sea shelves (about east of 5°E; **Supplementary Figure 6c**) and the center of the Norwegian Sea (about west of 5°E). The time-series associated with EOF-2 (**Supplementary Figure 6d**) corresponded to the inter-annual variability (**Supplementary Figure 7**). When summing the two first modes of variability, the dipole structure centered clearly on the AW path (**Supplementary Figure 6e**),

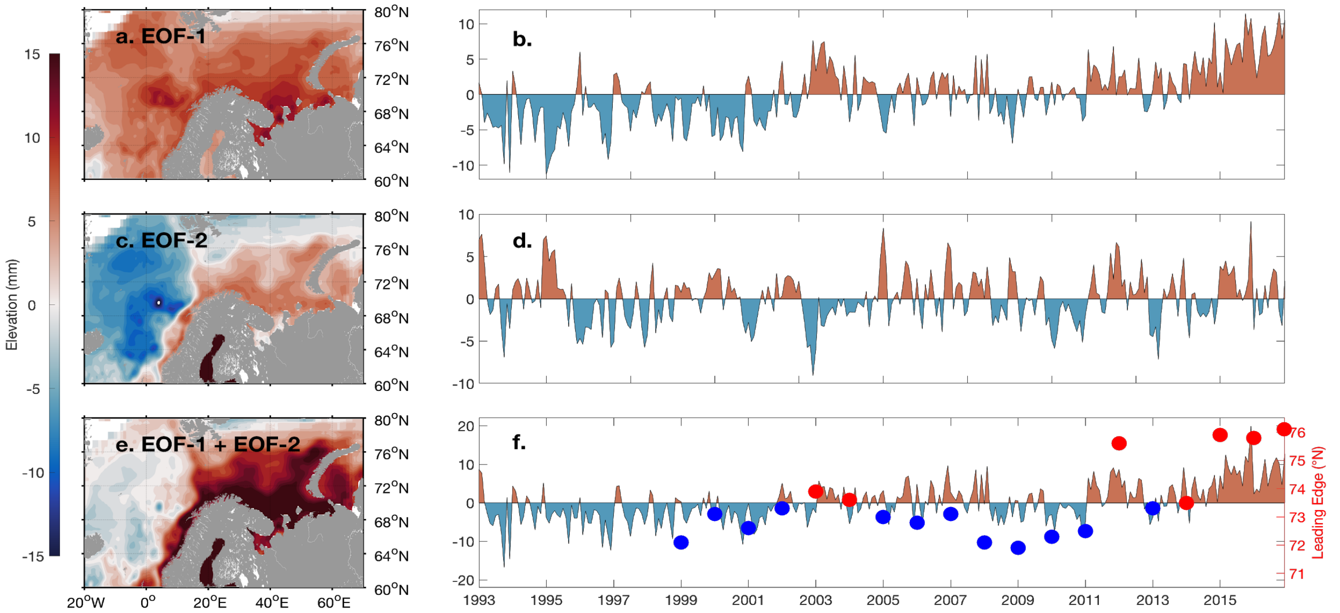

Supplementary Figure 6. First two modes (a and c) and associated time-series (b and d). Bottom panels (e and f) show the sum of the first two first EOF, which account for more than 80% of the total variability and display a positive linear trend. The blue/red dots correspond to the latitude of the leading-edge of the *Ehux* blooms in the eastern Barents Sea for longitudes > 40°E (right red y-axis), which strongly correlates with the yearly averaged EOF-1+EOF-2 time-series (Pearson's coefficient correlation r = 0.77, p = 0.0002, two-sided t-test).

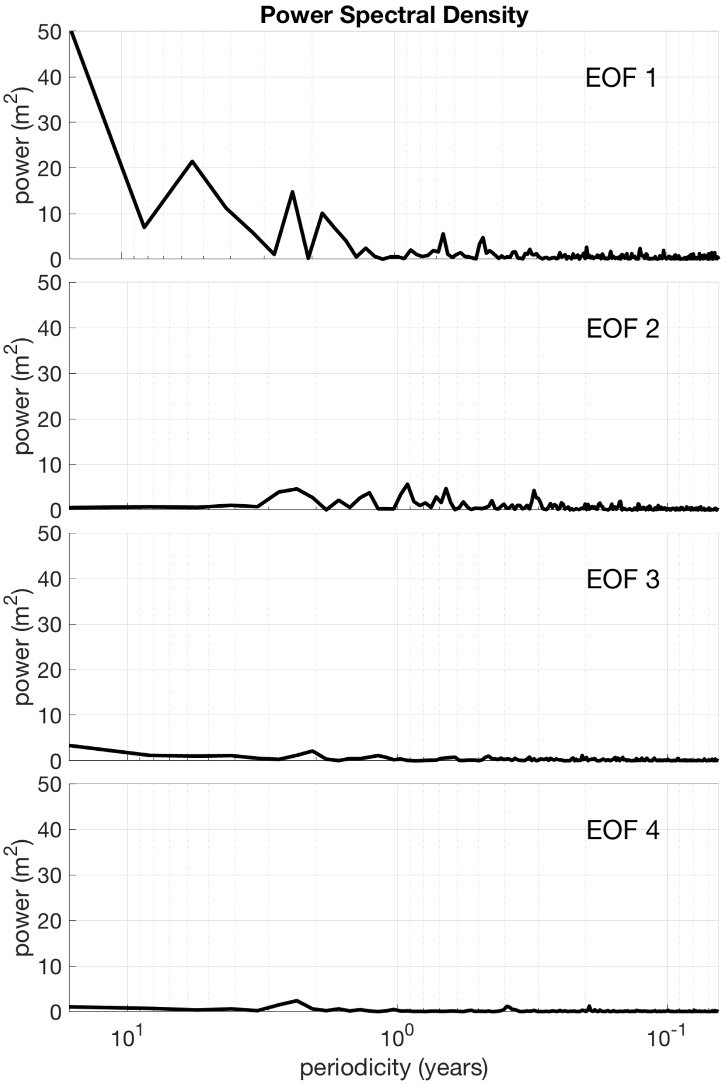

Supplementary Figure 7. Power spectrum of the four first EOF time-series.

To reveal the impact of changing sea level on surface velocity fields, recomposed Absolute Dynamic Topography (ADT) fields were then derived from the two first EOF modes (denoted as $ADT_{EOF1,2}$). Usually, ADT equals to the sum of MDT and SLA. To re-compose ADT maps corresponding to the positive and the negative phases of the EOF, we considered the extremums of the Principal Components (PC). For instance, the ADT_{EOF} at a time = t and position (*x0,y0*) is derived as: ADT_{EOF} (*t,x0,y0*) = MDT + EOF(*x0,y0*) x PC(*t*), where MDT is the mean dynamic topography, EOF is the spatial structure of each mode, and PC is the time-series associated to each mode. The obtained composite ADT_{EOF} fields were used to derive the associated composite surface absolute geostrophic velocities corresponding to the EOF using a 7-point pencil. The Matlab © code is provided (see code availability section). The corresponding surface absolute geostrophic velocities of the extreme negative (i.e. 1993) and positive (i.e. 2015) phases of the EOF are shown in **Supplementary Figure 8**. These two contrasting years illustrate the ongoing strengthening of the ocean surface circulation in the EAC. We show that changes in $ADT_{EOF1,2}$ were consistent with changes in ADT (i.e. the full signal as opposed to the signal associated to the first two modes only, **Supplementary Figure 8**), therefore confirming the robustness of the EOF analysis.

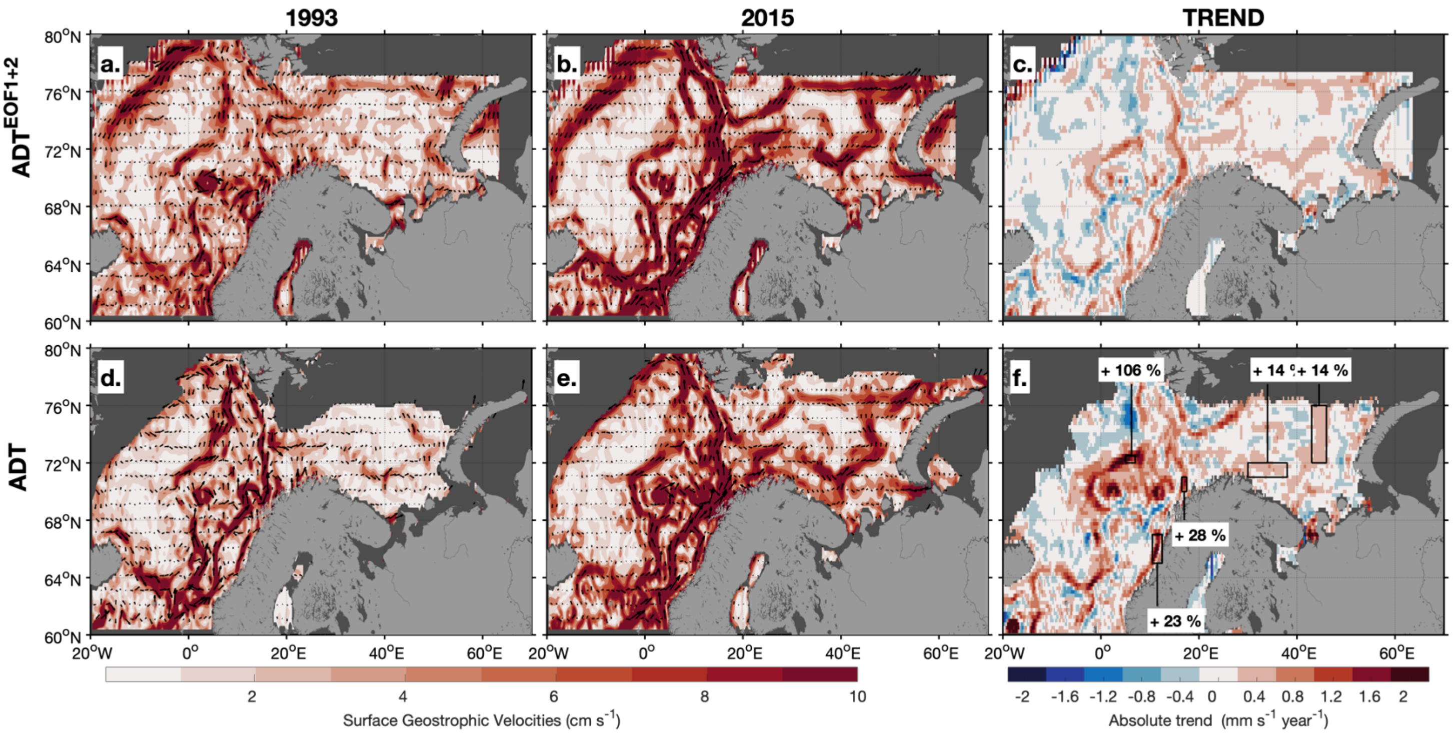

Supplementary Figure 8. Surface absolute geostrophic velocities derived from $ADT_{EOF1,2}$ (top) and ADT (bottom) fields during the extremums of the time-series which are respectively reached in December 1993 (a, d, minimum) and 2015 (b, e, maximum) with the corresponding absolute linear trend of the entire time-series (all months) over the 1993-2016 period (c, f). Areas covered by sea ice (sea ice concentration >15%) or with insufficient data coverage for the trend (< 50 %) are in dark gray. Note that for the re-composed ADT^{EOF}, the northern Barents Sea and the Kara Sea have been manually masked.

The current velocities are only expressed in absolute trends in the main text. We also show below the relative trend expressed in percentage only with significant pixels in **Supplementary Figure 9** ($p \leq 0.1$ two-sided t-test). Note that both velocity fields and the trends are derived from monthly composite maps of absolute geostrophic velocities. We remind that those monthly maps were derived from daily absolute geostrophic velocities directly provided by SL-TAC.

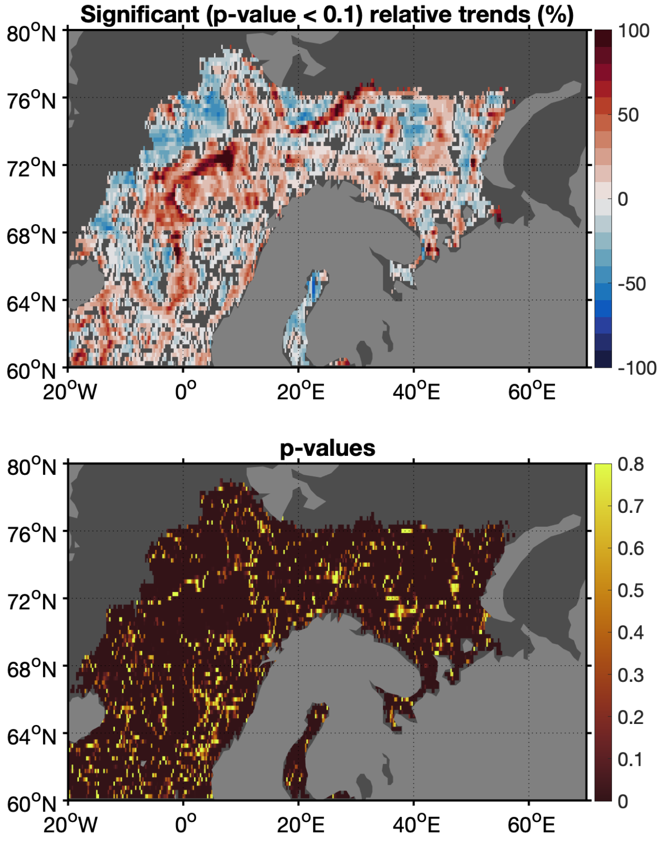

Supplementary Figure 9. (a) Relative trends (%) in current velocities for the EAC (%) associated with *p*-values < 0.05. Areas covered by sea ice (sea ice concentration > 15%), with insufficient data coverage for the trend (< 50 %) or non-significant trends ($p > 0.05$) are in dark gray. (b) map of *p*-values from the statistical two-sided *t*-test.

2.4. Robustness analysis of the Lagrangian experiments

Satellite-derived altimetry represents a valid quantity to obtain a close approximation of the real ocean current velocities. However, it presents some limitation, mainly due to the fact that it can capture only the geostrophic component of the velocity field. Ageostrophic and Ekman components are still not measurable through satellite observations. Furthermore, other effects such as the spatio-temporal variability in the sea surface height or instrumental errors can induce further imprecisions in the current estimation.

Despite all these limitations, recent studies confirmed the reliability of altimetric currents, in particular in the computation of Lagrangian diagnostics, under different uncertainty scenarios[16]. In addition, altimetry currents have already been used positively to infer temporal trends in current intensity, such as in the case of the Agulhas current[17,18].

Still, we decided to test the reliability of the satellite-derived velocity field used in our analyses to prove the robustness of the Lagrangian experiment. To do so, we performed an additional Lagrangian experiment (EXP.1d) in which we repeated EXP.1 (varying currents and temperature) 1000 times imposing a lateral turbulent diffusion coefficient D. This methodology, drawing inspiration from atmospheric Lagrangian analysis[19], has already been used to test the robustness of Lagrangian trajectories in the open ocean[20]. Using a diffusion D implies that, at

each time step of the advective process, each particle can move, in addition to the deterministic advection, of a distance delta along a random direction. We remind that[21]:

$$(1)\ D = \delta^2 \div (2 \times \tau)$$

where τ is the integration time step (in our case, 3 hours). In case of presence of an error on the velocity field δ_u, delta can be expressed as $\delta_u \times \tau$, which leads Eq (1) to:

$$(2)\ D = (\delta_u^2 \times \tau) \div 2$$

in which we illustrated the link between the diffusion coefficient and the error on the velocity field δ_u.

Estimation of the diffusion coefficient

From Eq. (2) it is possible to infer that it is necessary to know the error on the velocity field to obtain the diffusion coefficient. At present time, error maps for the velocity field do not exist (only an interpolation error map on SLA does). Therefore, we estimated δ_u with the methodology in the litterature[22]. This consists into using the standard deviation of a daily velocity field in the studied domain as error for that day. For each year, we computed the average standard deviation, which we transformed in equivalent diffusion using Eq. (2). Diffusion coefficients obtained in this way did not differ significantly from year to year, ranging between 26 and 30 $m^2\ s^{-1}$.
Complementarily, to test the reliability of such values, we used an experimental formal error, available for the global ocean for each day of 2017, at a resolution of 0.25 degree, processed by SSALTO/DUACS (Isabelle Pujol, pers. Comm.). We computed the mean formal error over our regions of interest, obtaining a value of δ_u = 0,041 m/s, which corresponds, using Eq. (2), to a diffusion coefficient of D = 9.2 m^2 s-*1*. A study on the temporal variability of the error showed that it did not change consistently across the 2017, ranging between 0,037 m s^{-1} (D = 7.5 $m^2\ s^{-1}$) and 0,045 m/s (D = 11 $m^2\ s^{-1}$). Only a small area of our domain (around Lofoten basin, between 0 and 13 degrees in longitude, and 68.8 and 70.2 of latitude) presented an error higher than the mean error, with δ_u = 0,064 m s^{-1}, corresponding to D = 22 $m^2\ s^{-1}$. This is why, for a more conservative and robust analysis, we decided to use the highest estimations of 30 $m^2\ s^{-1}$ for the diffusion coefficient D, in agreement with the existing literature[22].

Results of this Lagrangian experiment EXP.1d (with diffusion) are analyzed accordingly to the 'leading edge' method detailed in Supplementary method 1.3 and compared with the Lagrangian experiment EXP.1 (without diffusion). Results of this analysis are depicted in **Supplementary Figure 10** and showed that the expansion of the leading edge from EXP.1d is very similar to the non-diffusive scenario (trend = +380 kms vs +424 kms originally for EXP.1), with very low standard deviations. This realistic assessment of current error estimates evidences that the Lagrangian method is very robust to the addition of noise.

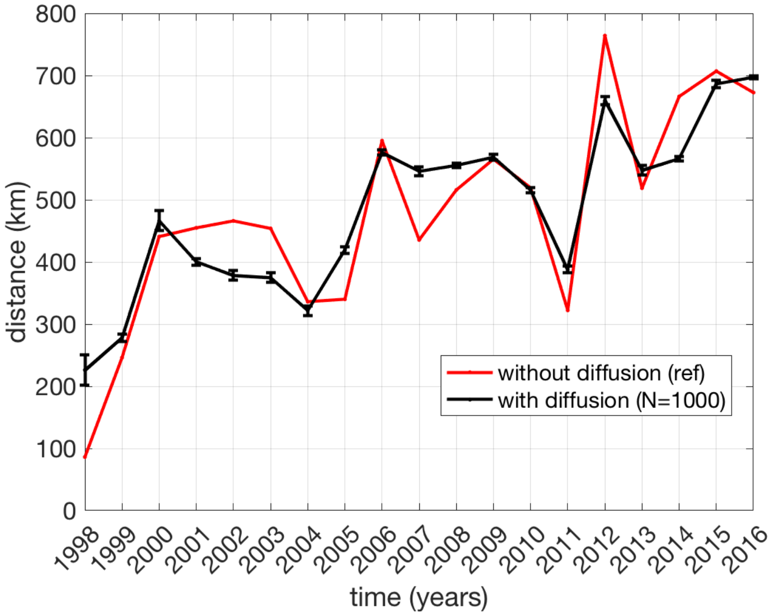

Supplementary Figure 10: Comparison between the 'leading edge' poleward expansion from EXP.1 (without diffusion, red) and EXP.1d (with diffusion, black). The results from EXP.1d are expressed as the mean (data points) and standard deviation (error bars) of 1000 experiments.

2.5. The *Ehux* minimal temperature for "regular" growth and delineation of the inoculum area

The temperature range for *Ehux* maximum growth rate is estimated to be around 21°C[23] with lower and upper boundaries of 10 and 26°C[24]. Below 10°C, the growth rate is not optimal anymore and is expected to decrease. The current largest collection of *Ehux* growth rates (laboratory data) demonstrated that the lowest temperature with a positive growth rate is at 4°C[25,26]. More recently, a model implemented from laboratory data indicated that the growth rate is becoming close to zero around 6°C[27]. However, laboratory studies may under-estimate actual *Ehux* growth rates at low temperatures. In the vicinity of the EAC, remote sensing studies observed different minimum temperature thresholds for the detection of *Ehux* blooms. This threshold was initially found to be 3°C[28], then updated[29] to 5°C and more recently defined as 6°C[1]. This temperature range is typical of Atlantic Waters[2]. On exceptional occasions, *Ehux* are even apt at surviving in polar conditions[30] (T ~ 0°C) but with expected low to very low growth rates. In the absence of consensus, and because almost no PIC is detected below 4°C in the EAC[29], we decided to choose for this study 4°C isotherm as threshold for the minimum temperature required for a "regular" growth that could allow the formation of a bloom. Below this limit, we consider that the *Ehux* development is not sustainable with either lower growth rate than losses or close to zero growth rate.

Most *Ehux* "hot-spots" are linked to shelves or shelf slopes[31–33], and, in the Norwegian Sea, summer *Ehux* abundances follow an east-west pattern characterized by increasing summer *Ehux* abundance toward the coast and with decreasing depth[34]. The Norwegian Fjords was proposed as the inoculum source for the entire region since *Ehux* bloom was observed there in early spring[32].

In order to use the most realistic simulation as possible, we decided to constrain the inoculum to the coastal area. The threshold of a maximum distance from the coast of about 180 km was based on the following leading-edge distance analysis. We derived the leading-edge

distance increase from the three Lagrangian experiments using an increasing maximum 'distance from the coast' threshold for the inoculum from 1 km to 400 km every 1 km (**Supplementary Figure 11**). We chose 180 km as a maximum 'distance from the coast' criterion to select virtual particles for the inoculum because it corresponded to the distance from which Lagrangian experiments 'compensated', i.e., when the increase in leading-edge distance from a Lagrangian experiment from varying temperature and currents (EXP1) equaled the sum of the increases in leading-edge derived from the constant temperature (EXP2) and the constant currents (EXP3) Lagrangian experiments. Results showed a good balance between the Lagrangian experiments within a 120-200 km range from the coast. If the threshold is smaller than 180 km, then the increase in leading-edge distance will be determined by EXP1 > EXP2 + EXP3. In the opposite scenario, if the threshold is greater than 180 km, then the increase in leading-edge distance will be determined by EXP1 < EXP2 +EXP3. When virtual particles are further than 200 km from the coast, the leading-edge distance from EXP3 increased abnormally compared to EXP1 which remains the reference, and EXP2+EXP3 exceed EXP1 by more than 10 %. When virtual particles are closer than 120 km from the coast, the leading-edge distance from EXP3 decreased abnormally compared to EXP1, and EXP2+EXP3 was more than 10% below EXP1.

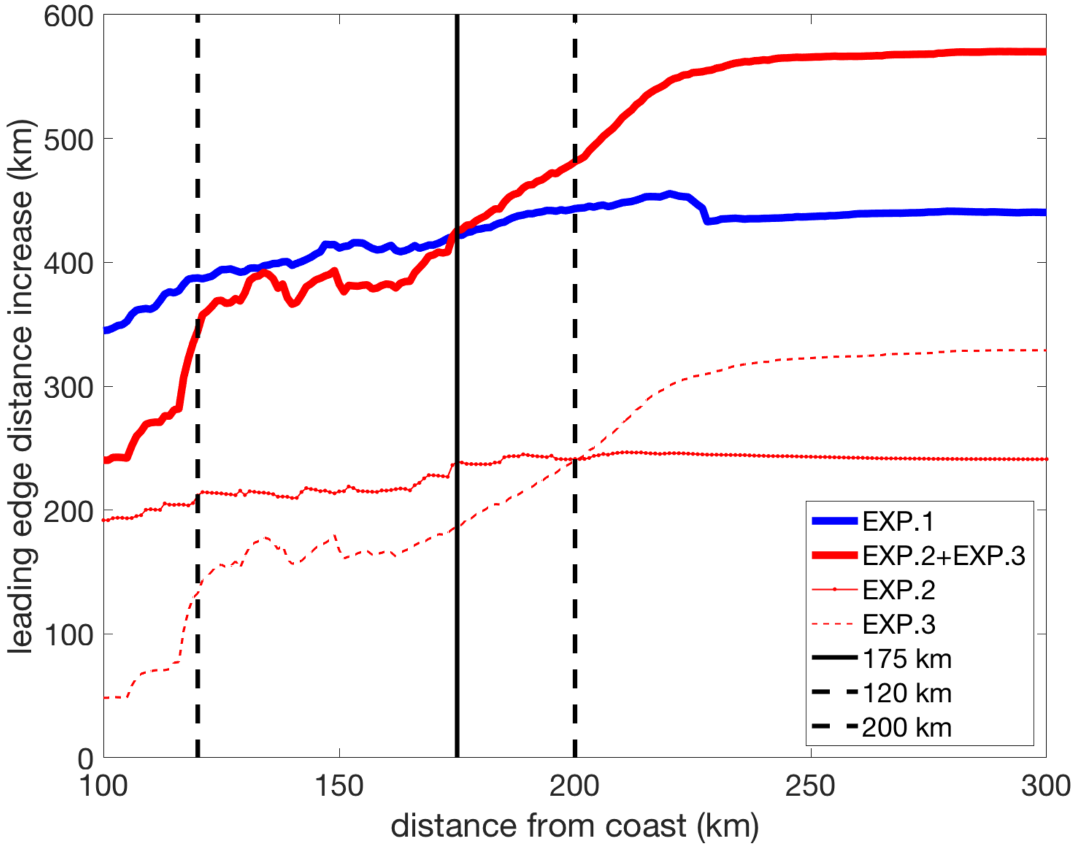

Supplementary Figure 11. Leading-edge distance (km) reached at the end of the Lagrangian experiments vs. maximum particles distance from the coast (km) at the start of the Lagrangian experiments (inoculum). The blue line is the 1st Lagrangian experiment (varying currents and temperature), while the red corresponds to the sum of the 2nd (constant temperature) and the 3rd (constant currents) Lagrangian experiments.

To make sure that the masking of virtual particles starting offshore (>180 km from the coast) did not alter the leading-edge detection in the eastern Barents Sea, we performed the following analysis. Virtual particles were binned at the end of their travel on a regular grid (0.25° in latitude and 0.5° in longitude) prior to their coastal (< 180 km) or basin (> 180 km) origins (**Supplementary Figure 12**). The analysis resulted, for an equal number of particles originating from both sides, in about 70% of particles ending up in the eastern Barents Sea displaying a coastal domain origin (i.e. < 180 km from coast). In other words, the particles which were located in the basin stayed in the deeper areas. This shows that masking particles, originated from very far from the coast, did not affect the leading-edge analyses in the eastern Barents Sea. In the eastern Barents Sea, most of the particles were originated for the coastal area.

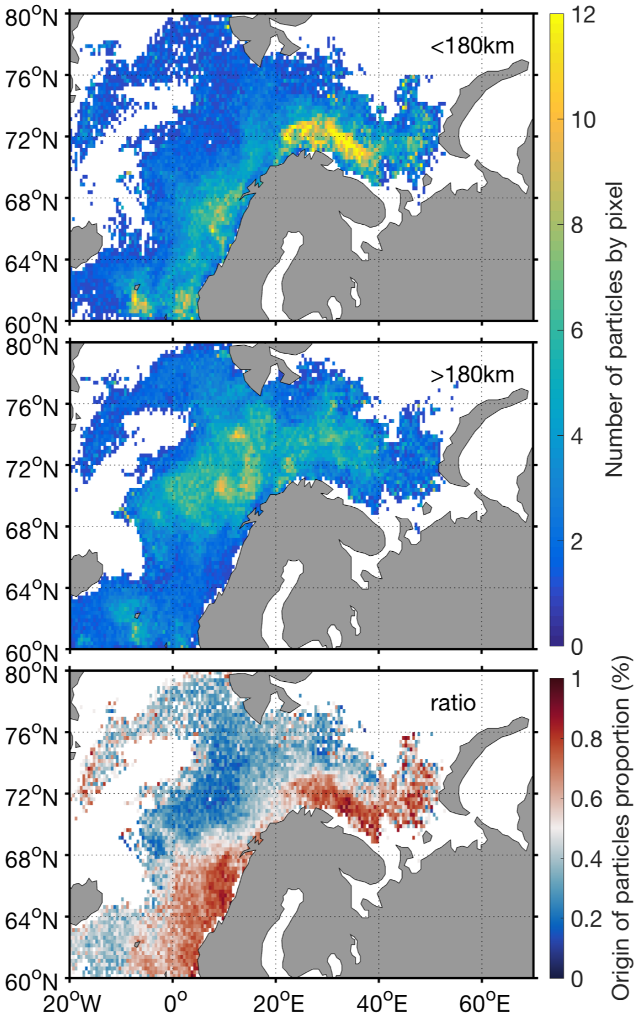

Supplementary Figure 12. Number of virtual particles by pixel at the end of the Lagrangian experiment (EXP1) originating from the coastal domain (top panel), the basin (middle panel) and their relative proportion (bottom). For the bottom panel, the red (blue) color indicates a ratio equal to 1 (0) and means that 100% (0%) of virtual particles originate from the coastal (basin) area.

2.6. Determination of the winter onset

In the following section, we discuss the choice to start the inoculum of parcels in March. March represents an extremum and an inflection point in term of Mixed Layer Depth (MLD; **Supplementary Figure 13**). During late March or early April, a drastic shallowing of the MLD (from 120 m to 60 m on average) is observed. At the same time, the photosynthetically available radiation (PAR) slowly starts to increase marking the beginning of the growing season for phytoplankton and allowing more favorable conditions particularly for *Ehux* in the southern Norwegian Sea and Nordic Seas. In the southern Norwegian Sea (blue box 1), the surface temperature stays above 4°C throughout the year, and thus would not be a limiting factor for *Ehux* growth. In the contrary, the northeastern Barents Sea (red box 2) is characterized by temperatures below 4°C from winter from November to July.

In the present work, we also assume that *Ehux* is able to migrate toward the Barents Sea carried by the Atlantic current. This advection starts as soon as *Ehux* is brought to the surface, during the transition from winter mixing to summer stratification. Indeed, coccolithophores are small (nano-) phytoplankton with low densities that restricts sinking. They can thus remain in surface or subsurface waters due to their large buoyancy. A sinking rate of about 0.26 m d^{-1} was estimated[24]. Therefore, a four-month travel from the Southern Norwegian Sea to the Barents Sea without vertical mixing would only make the phytoplankton sink to a maximum of about 30 m (in the hypohthesis the phytoplankton remains healthy). With vertical mixing, the phytoplankton is likely brought back at surface. In spring, the MLD is much deeper during March and April (>100 m on average, **Supplementary Figure 13**) and about the same order of magnitude in May and June (~30 and 20 m respectively, **Supplementary Figure 13**). Observations showed that the *Ehux* bloom peaked in May in the Fjords of southwest Norway[32], but it is likely that its growth rate increased before in the southern Norwegian Sea. Eventually, *Ehux* is likely to follow a poleward blooming succession, blooming in June in the Norwegian Sea and in July/August in the Barents Sea when temperatures exceed 4°C. During the rest of the year, temperatures much lower than this critical threshold of 4ºC combined with winter darkness and other top-down controls must prevent *Ehux* from year-to-year survival in the eastern Barents Sea.

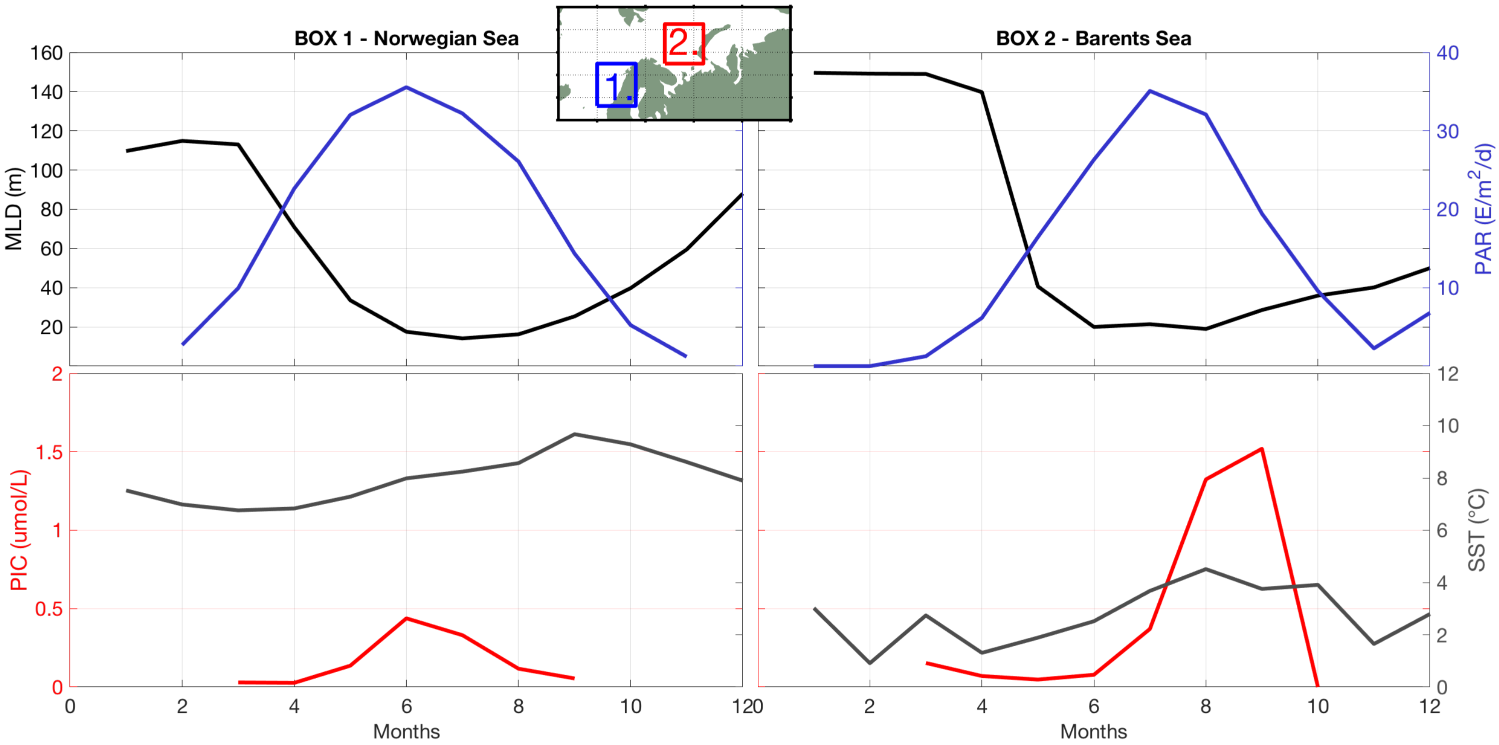

Supplementary Figure 13. Seasonal variation of environmental parameters in the Norwegian Sea (Box 1) and eastern Barents Sea (Box 2): MLD (m) climatologies (with density threshold criterion of 0.03 kg m^{-3} [35]) in blue, PAR (E m^{-2} d^{-1}) in black, PIC (µmol L^{-1}) in red (from MODIS climatologies) and in-situ SST (°C), in gray, from the Nordic Sea Atlas of the World Ocean DataBase[36].

2.7. Match-up between the 1st Lagrangian experiment and ocean color PIC

The First Lagrangian experiment (EXP1) was in very good agreement with the remotely sensed PIC (**Supplementary Figure 14**). We decided to quantify the matchups between particles ending up in the Barents Sea (> 20°E) and the summer PIC dataset. In order to improve data coverage, summer PIC was considered as the maximum concentration in the July-August-September period[1]. To do so, we counted the number of PIC pixels that were surrounded by at least one virtual particle in a 50 km radius perimeter (**Supplementary Figure 15**). Results of this analysis (**Supplementary Table 2**) show an 89% match-up for EXP1. Note that to avoid terrestrial contamination, PIC data corresponding to depth deeper than 80 m were systematically masked.

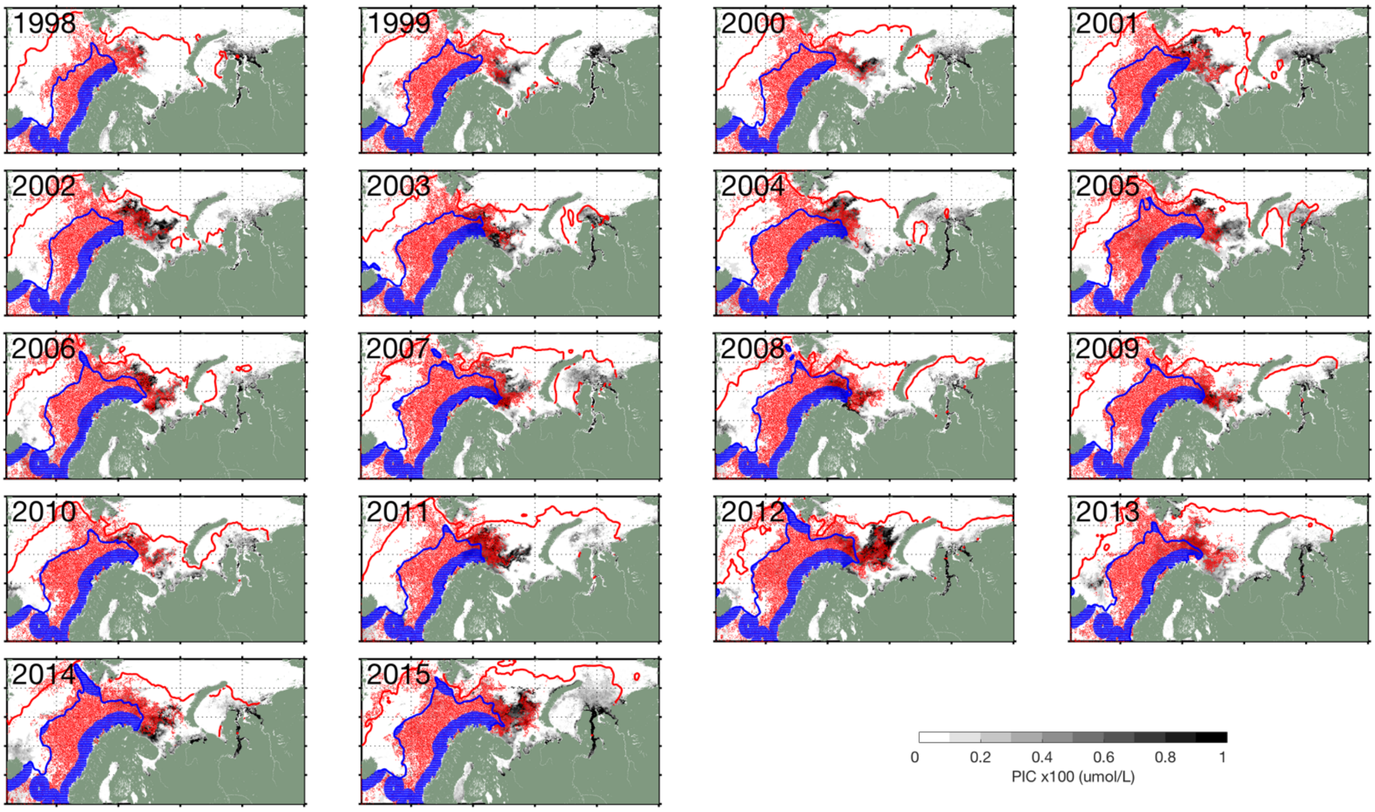

Supplementary Figure 14. Poleward expansion of *Emiliania Huxleyi* (*EHux*) in the European Arctic Corridor illustrated by the year-to-year evolution during the 1998-2016 period. The initialization (inoculum) of virtual particles in March (*Ehux*, blue dots) in the area defined by T ≥ 4 °C (March ecological niche, blue isoline). During six months, particles move with the currents as the ocean seasonally warms. In August, the particles end up in positions indicated by the red dots and the 4 °C isotherm is now indicated by the red line. In the background, remotely sensed PIC evidencing coccolithophore blooms in August in a gray palette.

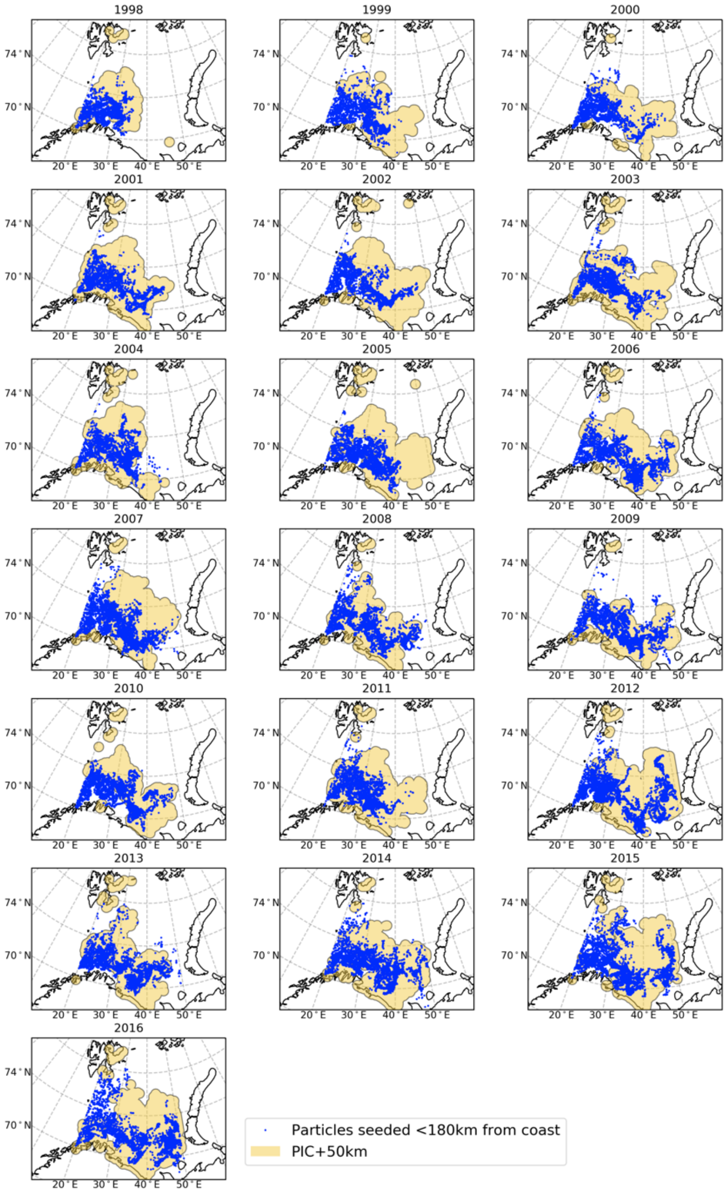

Supplementary Figure 15. Matchups between Lagrangian particles and ocean color PIC data. The red shading represents the bloom area, with a 50 km perimeter around PIC pixels exceeding 0.002 mol L^{-1}. Blue dots represent summer locations of all tracked particles at the end of the simulation. A virtual particle matched the PIC when it fell within the 50-km perimeter around a PIC value exceeding 0.002 mol L^{-1}.

Supplementary Table 2. Percentage of matchup between Lagrangian particles and ocean color PIC data through all years (1998-2016). A matchup was considered successful when a Lagrangian particle ended up in a position closer than 50 km from a PIC pixel exceeding 0.002 mol L^{-1}. Scores generally cluster around 70-90% for a critical distance of 50 km.

	EXP1	EXP2	EXP3
number of particles	1900	1900	1900
mean	80.9	73.0	78.3
Standard deviation	10.7	13.6	7.9
min	47.0	47.2	55.6
25% percentile	79.4	62.6	74.3
50% percentile	84.2	71.9	80.0
75% percentile	86.2	83.2	82.5
max	94.9	98.0	90.1

2.8. Linear trends and correlations statistics

Supplementary Table 3. Pearson's correlation coefficient and associated *p*-value (two-sided *t*-test) estimates and statistics of linear regression analyses of *Ehux* bloom leading-edge position over the time periods 1998-2016 and 2006-2016. Confidence intervals of 95% are given for Pearson's correlation coefficient r, and for the slope and offset of the linear regression, as dr, doffset and dslope, respectively.

dataset	r	dr	*p*	slope	offset	dslope	doffset
PIC (1998-2016)	0.569	0.329	0.0110	17.13	$-3.38e^4$	12.68	25436
PIC (2006-2016)	0.426	0.525	0.191	16.5	$-3.25e^4$	16.50	53055
EXP1 (1998-2016)	0.754	0.222	$1.91e^{-4}$	22.3	$-4.43e^4$	9.935	19940
EXP1 (2006-2016)	0.501	0.494	0.116	19.19	$-3.80e^4$	25.0	50225
EXP2 (1998-2016)	0.561	0.333	0.0125	12.62	$-2.50e^4$	9.55	19131
EXP2 (2006-2016)	0.732	0.345	0.0104	26.81	$-5.35e^4$	18.8	37840
EXP3 (1998-2016)	0.685	0.267	0.00121	9.824	$-1.928e^4$	5.35	10725
EXP3 (2006-2016)	0.101	0.596	0.769	0.764	-1052	0.765	11460

Supplementary Table 4. Pearson's correlation coefficients (and associated *p*-values from two-sided *t*-test) of the relationships between leading-edge distance derived from the different Lagrangian experiments and from the Ocean Color PIC dataset (1998-2016).

**r (*p*-value)**	derived from PIC	EXP1	EXP2	EXP3
PIC	1			
EXP1	0.7544 (0.0002)	1		
EXP2	0.7104 (0.0007)	0.7580 (0.0002)	1	
EXP3	0.4348 (0.0629)	0.5998 (0.0066)	0.2392 (0.3239)	1

2.9. The mean 'distance travelled' by particles

For the 2nd and 3rd Lagrangian experiments (EXP2 and (EXP3), the distance travelled for each virtual particle was derived. Statistics pertaining to the particles entering the Barents Sea revealed that the average distance travelled by particles significantly increased by 113 km during EXP2 and decreased by 30 km during EXP3 (**Supplementary Figure 16**).

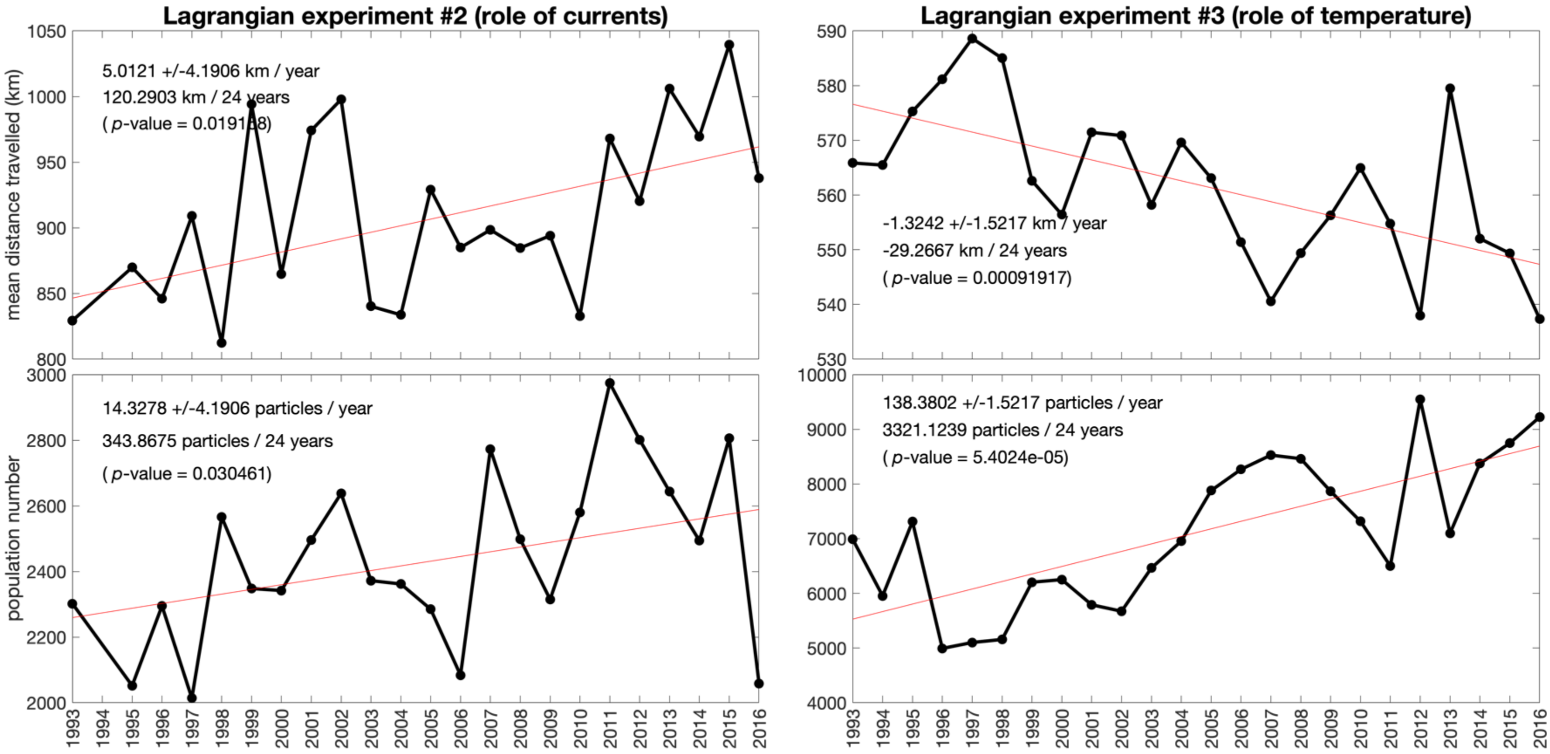

Supplementary Figure 16. Linear trend (least-squares regression) and associated *p*-values (two-sided *t*-test) in (a) mean distance travelled by virtual particules for EXP2 and (b) EXP3 (b); (c) number of Lagrangian particles for EXP2 and (d) EXP3 (d) entering the Barents Sea (longitude > 20°E) and originating from the inoculum area ($SST_{march} \geq 4°C$ and distance from coast ≤ 180 km).

2.10 Increase of the *Ehux* bloom surface area

The *Ehux* bloom expansion is also reflected in its increased surface area which doubled between 1998 and 2016 (**Supplementary Figure 17**). Here, we derived the linear trend associated with the yearly increase in the summer *Ehux* bloom area in the Barents Sea considering a PIC threshold of 0.002 mol L^{-1}. The increase corresponded to an 83313 km^2 increase of the bloom surface area (+88.8 %). The bloom increased from 10.2% in term of Barents Sea surface occupation in summer to up to 19 %.

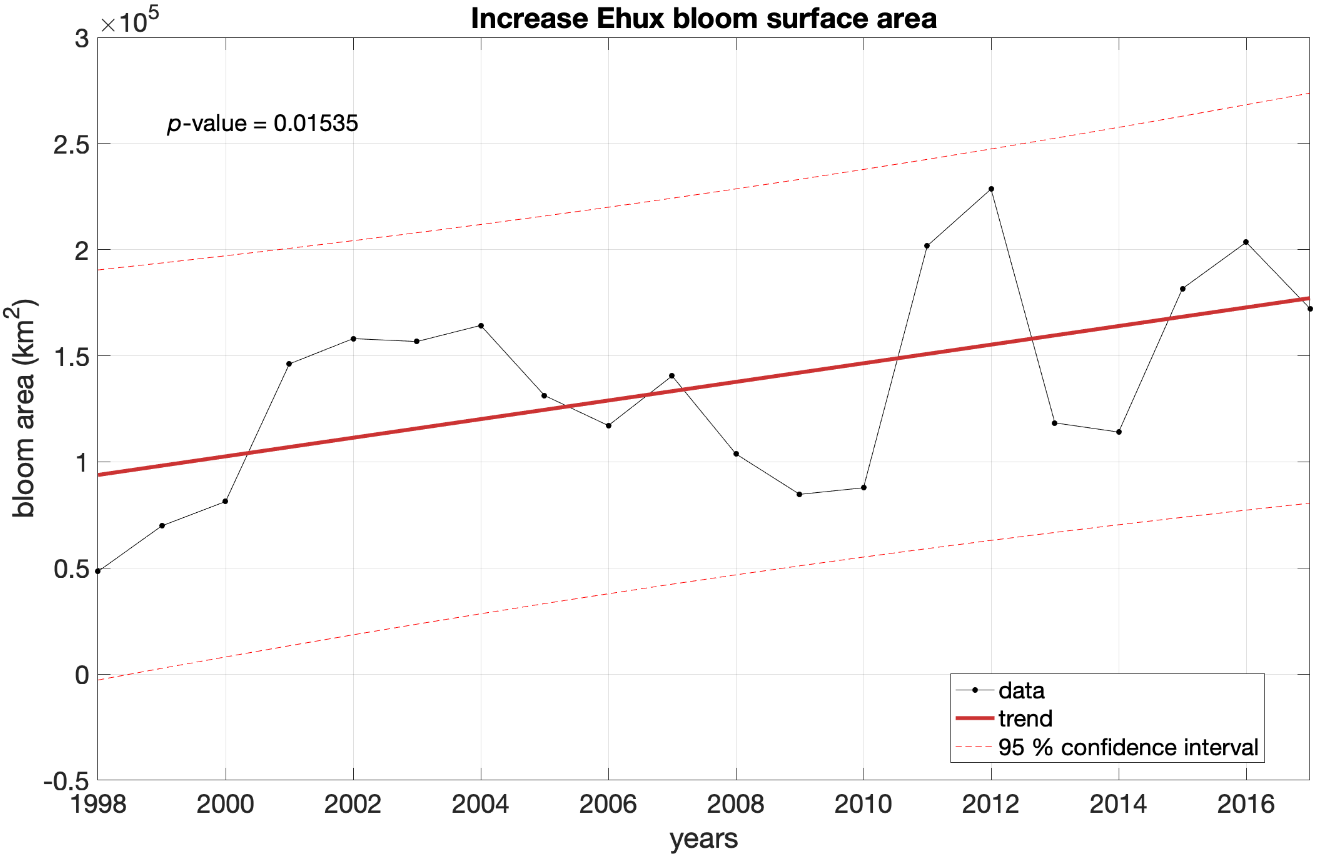

Supplementary Figure 17. Linear trend (least-squares regression) of *Ehux* bloom surface area between 1998 and 2016 and its associated *p*-value and 95 % confidence bounds (derived with a two-sided *t*-test).

References

1. Neukermans, G., Oziel, L. & Babin, M. Increased intrusion of warming Atlantic water leads to rapid expansion of temperate phytoplankton in the Arctic. *Glob. Chang. Biol.* **24**, 2545–2553 (2018).
2. Oziel, L., Sirven, J. & Gascard, J. C. The Barents Sea frontal zones and water masses variability (1980-2011). *Ocean Sci.* **12**, 169–184 (2016).
3. Oziel, L. et al. Role for Atlantic inflows and sea ice loss on shifting phytoplankton blooms in the Barents Sea. *J. Geophys. Res. Ocean.* **122**, 5121–5139 (2017).
4. Ingvaldsen, R. B., Asplin, L. & Loeng, H. The seasonal cycle in the Atlantic transport to the Barents Sea during the years 1997-2001. *Cont. Shelf Res.* **24**, 1015–1032 (2004).
5. Raj, R. P. et al. Quantifying Atlantic Water transport to the Nordic Seas by remote sensing. *Remote Sens. Environ.* **216**, 758–769 (2018).
6. Lien, V. S., Vikebø, F. B. & Skagseth, Oø. One mechanism contributing to co-variability of the Atlantic inflow branches to the Arctic. *Nat. Commun.* **4**, 1488 (2013).
7. Lien, V. S., Schlichtholz, P., Skagseth, Ø. & Vikebø, F. B. Wind-Driven Atlantic Water Flow as a Direct Mode for Reduced Barents Sea Ice Cover. *J. Clim.* **30**, 803–812 (2016).
8. Ingvaldsen, R. B., Asplin, L. & Loeng, H. Velocity field of the western entrance to the Barents Sea. *J. Geophys. Res. Ocean.* **109**, 1–12 (2004).
9. Skagseth, Ø. Recirculation of Atlantic Water in the western Barents Sea. *Geophys. Res. Lett.* **35**, 1–5 (2008).
10. Mork, K. A. & Skagseth. A quantitative description of the Norwegian Atlantic Current by combining altimetry and hydrography. *Ocean Sci.* **6**, 901–911 (2010).
11. Liu, Y., Weisberg, R. H., Vignudelli, S., Roblou, L. & Merz, C. R. Comparison of the X-TRACK altimetry estimated currents with moored ADCP and HF radar observations on the West Florida Shelf. *Adv. Sp. Res.* **50**, 1085–1098 (2012).
12. Cipollini P. et al. Climate-quality estimates of sea level in the coastal zone from the ESA Climate Change Initiative Sea Level Project. in *8th Coastal Altimetry Workshop -* **CAW-8** (2014).
13. Legeais, J.-F. et al. An improved and homogeneous altimeter sea level record from the ESA Climate Change Initiative. *Earth Syst. Sci. Data* **10**, 281–301 (2018).
14. Vignudelli, S., Kostianoy, A. G., Cipollini, P. & Benveniste, J. *Coastal altimetry*. 389–413 (2011).
15. Volkov, D. L., Landerer, F. W. & Kirillov, S. A. The genesis of sea level variability in the Barents Sea. *Cont. Shelf Res.* **66**, 92–104 (2013).
16. Hernández-Carrasco, I., López, C., Hernández-García, E. & Turiel, A. How reliable are finite-size Lyapunov exponents for the assessment of ocean dynamics? *Ocean Model.* **36**, 208–218 (2011).
17. Backeberg, B. C., Penven, P. & Rouault, M. Impact of intensified Indian Ocean winds on mesoscale variability in the Agulhas system. *Nat. Clim. Chang.* **2**, 608–612 (2012).
18. Elipot, S., Beal, L. M., Elipot, S. & Beal, L. M. Observed Agulhas Current sensitivity to interannual and long-term trend atmospheric forcings. *J. Climate,* **31***,* 3077–3098 (2018).
19. Legras, B., Pisso, I., Berthet, G. & Lefèvre, F. Variability of the Lagrangian turbulent diffusion in the lower stratosphere. *Atmos. Chem. Phys.* **5**, 1605–1622 (2005).
20. Cotte, C. et al. Scale-dependent interactions of Mediterranean whales with marine dynamics. *Limnol. Oceanogr.* **56**, 219–232 (2011).
21. Berg, H. C. Random walks in biology. (Princeton University Press, 1993).
22. Leeuwenburgh, O. & Stammer, D. Uncertainties in altimetry-based velocity estimates.

J. Geophys. Res. 107, 3175 (2002).
23. Brun, P. et al. Ecological niches of open ocean phytoplankton taxa. *Limnol. Oceanogr.* **60**, 1020–1038 (2015).
24. Paasche, E. A review of the coccolithophorid *Emiliania huxleyi* (Prymnesiophyceae), with particular reference to growth, coccolith formation, and calcification-photosynthesis interactions. *Phycologia* **40**, 503–529 (2001).
25. Fielding, S. R. *Emiliania huxleyi* specific growth rate dependence on temperature. *Limnol. Oceanogr*. **58**, 663–666 (2013).
26. Krumhardt, K. M., Lovenduski, N. S., Freeman, N. M. & Bates, N. R. Apparent increase in coccolithophore abundance in the subtropical North Atlantic from 1990 to 2014. *Biogeosciences* **13**, 1163–1177 (2016).
27. Wang, X. et al. How will the key marine calcifier *Emiliania huxleyi* respond to a warmer and more thermally variable ocean? *Biogeosciences* **16**, 4393–4409 (2019).
28. Iglesias-Rodríguez, M. D. et al. Representing key phytoplankton functional groups in ocean carbon cycle models: Coccolithophorids. **Global Biogeochem. Cycles** *16*, 47-1-47–20 (2002).
29. Signorini, S. R. & McClain, C. R. Environmental factors controlling the Barents Sea spring-summer phytoplankton blooms. *Geophys. Res. Lett*. **36**, 1–5 (2009).
30. Hegseth, E. N. & Sundfjord, A. Intrusion and blooming of Atlantic phytoplankton species in the high Arctic. *J. Mar. Syst*. **74**, 108–119 (2008).
31. Brown, C. W. & Yoder, J. A. Coccolithophorid blooms in the global ocean blooms annually covered an average. *Atlantic* **99**, 7467–7482 (1994).
32. Tyrrell, T. & Merico, A. *Emiliania huxleyi*: bloom observations and the conditions that induce them. Coccolithophores 75–97 (2004).
33. Hopkins, J., Henson, S. A., Painter, S. C., Tyrrell, T. & Poulton, A. J. Phenological characteristics of global coccolithophore blooms. *Global Biogeochem. Cycles* **29**, 239–253 (2015).
34. Dylmer, C. V., Giraudeau, J., Hanquiez, V. & Husum, K. The coccolithophores *Emiliania huxleyi* and *Coccolithus pelagicus*: Extant populations from the Norwegian-Iceland Seas and Fram Strait. *Deep. Res. Part I Oceanogr. Res. Pap*. **98**, 1–9 (2015).
35. de Boyer Montégut, C., Madec, G., Fischer, A. S., Lazar, A. & Iudicone, D. Mixed layer depth over the global ocean: An examination of profile data and a profile-based climatology. *J. Geophys. Res. C Ocean*. **109**, 1–20 (2004).
36. Korablev, A. A., Smirnov, A. V. & Baranova, O. K. Climatological atlas of the Nordic Seas and Northern North Atlantic. *NOAA Atlas* NESDIS **77** 13, 116 (2014).
