# Supplementary material for: Faster Atlantic currents drive poleward expansion of temperate phytoplankton in the Arctic Ocean
Source: Nat Commun. 2020 Apr 6;11:1705. doi: 10.1038/s41467-020-15485-5 (PMC7136244; doi:10.1038/s41467-020-15485-5)
Supplement: Supplementary file 3 — Reporting Summary [file 41467_2020_15485_MOESM3_ESM.pdf]

nature research

Corresponding author(s): Laurent Oziel

Last updated by author(s): Feb 14, 2020

Reporting Summary

Nature Research wishes to improve the reproducibility of the work that we publish. This form provides structure for consistency and transparency in reporting. For further information on Nature Research policies, see Authors & Referees and the Editorial Policy Checklist .

Statistics

For all statistical analyses, confirm that the following items are present in the figure legend, table legend, main text, or Methods section.

n/a	Confirmed	
☐	☒	The exact sample size (*n*) for each experimental group/condition, given as a discrete number and unit of measurement
☐	☒	A statement on whether measurements were taken from distinct samples or whether the same sample was measured repeatedly
☐	☒	The statistical test(s) used AND whether they are one- or two-sided<br>*Only common tests should be described solely by name; describe more complex techniques in the Methods section.*
☒	☐	A description of all covariates tested
☒	☐	A description of any assumptions or corrections, such as tests of normality and adjustment for multiple comparisons
☐	☒	A full description of the statistical parameters including central tendency (e.g. means) or other basic estimates (e.g. regression coefficient) AND variation (e.g. standard deviation) or associated estimates of uncertainty (e.g. confidence intervals)
☐	☒	For null hypothesis testing, the test statistic (e.g. *F*, *t*, *r*) with confidence intervals, effect sizes, degrees of freedom and *P* value noted<br>*Give P values as exact values whenever suitable.*
☒	☐	For Bayesian analysis, information on the choice of priors and Markov chain Monte Carlo settings
☒	☐	For hierarchical and complex designs, identification of the appropriate level for tests and full reporting of outcomes
☐	☒	Estimates of effect sizes (e.g. Cohen's *d*, Pearson's *r*), indicating how they were calculated

Our web collection on statistics for biologists contains articles on many of the points above.

Software and code

Policy information about availability of computer code

Data collection	No software was used for data collection.
Data analysis	The data was analyzed with custom MatLab 2018a (c) codes. Both codes and processed data that will be publicly available before publication on the following gitlat depository https://gitlab.com/loloziel/oziel_et_al_nc_2020

For manuscripts utilizing custom algorithms or software that are central to the research but not yet described in published literature, software must be made available to editors/reviewers. We strongly encourage code deposition in a community repository (e.g. GitHub). See the Nature Research guidelines for submitting code & software for further information.

Data

Policy information about availability of data

All manuscripts must include a data availability statement. This statement should provide the following information, where applicable:

- Accession codes, unique identifiers, or web links for publicly available datasets
- A list of figures that have associated raw data
- A description of any restrictions on data availability

All the data used in this research are freely available to the public and may be downloaded through the following links: http://hermes.acri.fr (PIC ocean color satellite data), http://marine.copernicus.eu/ (product ID: SEALEVEL_GLO_PHY_L4_REP_OBSERVATIONS_008_047 for altimetric data), and https://www.esrl.noaa.gov/psd/data/gridded/data.noaa.oisst.v2.highres.html (for SST data). Analysis outputs data for the main figures will be made available at https://gitlab.com/loloziel/oziel_et_al_nc_2020 before publication. The depository will be populated before sending typesetting corrections.

Field-specific reporting

Please select the one below that is the best fit for your research. If you are not sure, read the appropriate sections before making your selection.

☐ Life sciences ☐ Behavioural & social sciences ☒ Ecological, evolutionary & environmental sciences

For a reference copy of the document with all sections, see nature.com/documents/nr-reporting-summary-flat.pdf

Ecological, evolutionary & environmental sciences study design

All studies must disclose on these points even when the disclosure is negative.

Study description	This study is based on satellite data (Ocean color, altimetry,microwave SST or sea ice concentration). All data is available freely. The analysis consist in EOF, trends, correlations of time series. There was also 3 Lagrangian experiments which robustness was validated using a 'Monte-Carlo' analysis repeating 1000 times the experiment.
Research sample	This study is based on satellite PIC ocean color data (Globcolor http://hermes.acri.fr), Altimetric data (http://marine.copernicus.eu/ ; product ID: SEALEVEL_GLO_PHY_L4_REP_OBSERVATIONS_008_047), sea surface temperature and sea ice concentration data (https://www.esrl.noaa.gov/psd/data/gridded/data.noaa.oisst.v2.highres.html).
Sampling strategy	This does not apply to our study which is based exclusively on satellite data.
Data collection	The data collection is out of the scope of the paper since it is satellite data. In our case, the collection is limited to the download of freely available satellite data. The data set are 'merged' multi-mission products that involves dozens of different satellites and sensors deployed by several space agencies over the past 20 years. The reader can refer to the detailed method section to know more about the considered sensors and/or the online product's documentation for more informations about their specificities.
Timing and spatial scale	In this study, we considered time series from 1993-2016 for satellite altimetry data and 1998-2016 for ocean color satellite data. We used monthly composite maps except for the Lagrangian experiments that used daily fields of geostrophic velocities directly from CMEMS (see method)
Data exclusions	In the process of building monthly composite maps, we post-processed data to exclude data associated with sea ice (sea ice concentration>15%) and large error estimates (>50% of the signal variance) in order to select only reliable data. For the leading-edge detection, we employed a custom mask for the eastern Barents Sea that we make available.
Reproducibility	The reproducibility is straightforward and must not lead to different result.
Randomization	This does not apply to our study.
Blinding	This does not apply to our study.

Did the study involve field work? ☐ Yes ☒ No

Reporting for specific materials, systems and methods

We require information from authors about some types of materials, experimental systems and methods used in many studies. Here, indicate whether each material, system or method listed is relevant to your study. If you are not sure if a list item applies to your research, read the appropriate section before selecting a response.

Materials & experimental systems

n/a	Involved in the study	
☒	☐	Antibodies
☒	☐	Eukaryotic cell lines
☒	☐	Palaeontology
☒	☐	Animals and other organisms
☒	☐	Human research participants
☒	☐	Clinical data

Methods

n/a	Involved in the study	
☒	☐	ChIP-seq
☒	☐	Flow cytometry
☒	☐	MRI-based neuroimaging
